# Eclectic Ocular Comorbidities and Systemic Diseases with Eye Involvement: A Review

**DOI:** 10.1155/2016/6215745

**Published:** 2016-03-14

**Authors:** María D. Pinazo-Durán, Vicente Zanón-Moreno, José J. García-Medina, J. Fernando Arévalo, Roberto Gallego-Pinazo, Carlo Nucci

**Affiliations:** ^1^Ophthalmic Research Unit “Santiago Grisolía”, Fundación Investigación Biomédica y Sanitaria (FISABIO), Avenida Gaspar Aguilar 90, 46017 Valencia, Spain; ^2^Ophthalmology Research Unit, Department of Surgery, Faculty of Medicine and Odontology, University of Valencia, Avenida Blasco Ibáñez 15, 46010 Valencia, Spain; ^3^Spanish Net of Ophthalmic Pathology (OFTARED), Instituto de Salud Carlos III, C/Sinesio Delgado 4, 28029 Madrid, Spain; ^4^Ophthalmology Department, University Hospital Reina Sofia, Avenida Intendente Jorge Palacios 1, 30003 Murcia, Spain; ^5^Retina Division, The Wilmer Eye Institute, The Johns Hopkins University School of Medicine, Nelson Building, 600 N. Wolf Street, Baltimore, MD 21287, USA; ^6^Department of Ophthalmology, University and Polytechnic Hospital La Fe, Avenida Fernando Abril Martorell 106, 46026 Valencia, Spain; ^7^Ophthalmic Unit, Department of Experimental Medicine and Surgery, University of Rome Tor Vergata, 81 Oxford Street, 00133 Rome, Italy

## Abstract

Coexistence of several ocular diseases is more frequent than suspected. In spite of the refractive errors, one or more of the following can be detected simultaneously: glaucoma, cataracts, uveitis, age-related macular degeneration, and dry eyes. In addition, as people age, ocular comorbidities are much more usually seen. Specific diseases are openly acknowledged to affect the eyes and vision, such as diabetes mellitus, hypertension blood pressure, arthritis, hyperthyroidism, neurodegenerative disorders, hematologic malignancies, and/or systemic infections. Recent advances in early diagnosis and therapy of the ophthalmic pathologies have reinforced patient options to prevent visual impairment and blindness. Because of this, it is essential not to overlook sight-threatening conditions such as the ocular comorbidities and/or the eye involvement in the context of systemic disorders. Moreover, the important role of the multidisciplinary cooperation to improve and sustain management of patients affected with eclectic ocular comorbidities and/or systemic disorders with eye repercussion is specifically addressed. This review intends to shed light on these topics to help in making opportune diagnosis and appropriately managing the affected patients.

## 1. Introduction

Currently, “comorbid” is employed to define a medical process that simultaneously exists in a patient with one or more medical conditions that, in turn, are independent themselves. In ophthalmology, “ocular comorbidities” are the eye disorder combinations existing simultaneously regardless of their etiopathogenic relationship [1–3]. This condition requires an effort from the ophthalmologist to gather information about the patients' morbidity. In fact, clusters of eye diseases/disorders composing the comorbidity patterns have to be necessarily identified. As it is quite rightly recognized, the eye importantly contributes to the diagnosis of a wide variety of systemic disorders, many times being the first visible clinical manifestation of the general problem, as well [4–6]. Early diagnosis and therapy may help anticipate or avoid complications.

**Figure 1:**
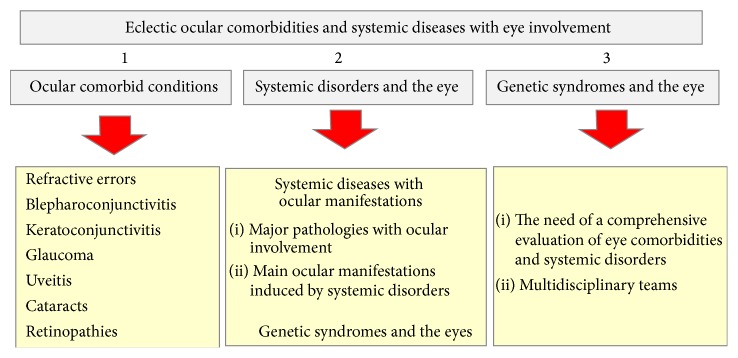
Flow chart on the distinct sections included in this review.

Because of the importance of these topics, in this work, we have looked at some particular ocular conditions and also at the most relevant systemic disorders that may affect the eye, and that has been also considered constituting a pose challenge to vision.  Figure 1 is a flow chart of the distinct sections considered in this review.

## 2. Ocular Comorbid Conditions

The coincidence of two diseases is not the simple “arithmetic” addition of both processes, while this condition also creates a new status in the eye health that obviously warrants new consideration and outstanding strategies.

An overview of the most commonly seen associations in the clinical practice includes refractive errors and other eye diseases, anterior eye segment and adnexa processes such as conjunctivitis/keratitis/blepharitis, and ocular surface disorders with uveitis, cataracts, glaucoma, diabetic retinopathy (DR), or age-related macular degeneration (AMD), as reflected in Figure 1. Better knowledge of the ocular comorbidities is important to achieve a more accurate diagnosis and therapy of these disorders. Some of them are exposed in further detail below.

Searching the scientific literature, the most eclectic ocular comorbidities with high refractive errors have to be considered in the context of the three processes: (1) astigmatism, (2) hyperopia, and (3) myopia, all of them coexisting or not with anisometropia. A recent study carried out on 137 keratoconic patients concluded that 65% displayed with-the-rule anterior corneal astigmatism and 80% of eyes had against-the-rule posterior corneal astigmatism [7]. Fleischer ring, prominent corneal nerves, and corneal thinning have been recently described in association with typical keratoconus manifestations, like in asymptomatic individuals [8]. It is widely recognized that children with higher hyperopia are likely to display strabismus, amblyopia, and poor stereopsis, but other systemic disorders and/or developmental abnormalities have also been reported [9]. Higher myopia is usually associated with eye diseases such as glaucoma, cataracts, and choroidal neovascularization, significantly contributing to augmenting the risk of visual impairment and blindness in these patients [10]. Other eye comorbidities have been described in cases of advanced surface ablation during laser refractive surgery that manifested themselves in the postoperative period with signs and symptoms such as burning/foreign body sensation, tearing, pain, and photophobia. As a consequence of this, instauration of a precise analgesic protocol has been recently suggested for patients subjected to these procedures [11].

Dry eye disease (DED) is a multifactorial disorder affecting the integrity of the lacrimal functional unit that frequently appears discordantly with the signs and symptoms [12]. Usually DEDs are linked to other eye diseases. Dry eyes have been found to be associated with vernal keratoconjunctivitis in children, probably affecting the ocular surface also during the quiescent phases of the disease. This finding contributes to our understanding of the very long-term consequences of this and other similar chronic mechanisms potentially damaging the ocular surface [13]. In analogous manner, DEDs of various degrees of severity have been reported in glaucoma patients chronically using hypotensive eye drops [14].

Uveitis is an important disease for the numerous eye complications that may occur in children and adults, many of which are vision threatening. It is known that these drawbacks increase with duration of disease. Specifically, noninfectious uveitis results in vision loss and a variety of ocular complications without adequate treatment. When comparing the risk of developing ocular complications between patients with noninfectious intermediate uveitis, posterior uveitis, or panuveitis, Dick et al. [15] reported that particular persistent cases are strongly associated with a higher risk of ocular comorbid complications (including band keratopathy, cataracts, glaucoma, and/or cystoid macular edema) than the matched healthy controls.

The number of people with cataracts has been estimated to increase (to 30.1 million and to reach 2.95 million, resp.) by 2020. Cataracts are the first evitable cause of blindness in the world. In general, cataracts are linked to aging. However, some cataracts can be related to genetic disorders, systemic diseases, long-term use of specific medications, or other eye conditions, such as uveitis, aniridia, glaucoma, ocular traumatisms, retinal detachment, retinopathy of prematurity, retinitis pigmentosa, DR, or AMD. Among the risk factors for cataract development or progression, the following should be considered: DM, tobacco and alcohol habits, prolonged exposure to sunlight, corticosteroid systemic or local therapy, electric and heat injuries, nutritional facts, and so forth, some of them remaining controversial [16]. A study on the ocular comorbidities among 313 cataract-operated patients in rural China revealed that the leading comorbidities were the presence of refractive error (60%) followed by glaucoma (19%) [17]. And in this context, new surgical devices have been arising for implementing the results of the combined cataract and glaucoma interventions [18]. Higher demand of cataract surgery worldwide and the resulting complications need the instauration of outstanding strategies for avoiding vision loss. Different prophylactic measures have been recently reviewed to prevent macular edema after phacoemulsification surgery [19].

Glaucoma is the first cause of irreversible blindness worldwide. Major risk factor is the increased and sustained intraocular pressure (IOP) that induces optic nerve degeneration and atrophy [20]. There are two main glaucoma types, the open-angle (POAG) and the closed-angle (PCAG), the first being the most frequent clinical glaucoma form. It is well known that several hereditary conditions are associated with glaucoma, but other causes include prolonged use of corticosteroids, vascular abnormalities, and reduced blood flow to the eyeballs (as in the course of DR or retinal vascular occlusions) [21]. Correspondingly, ocular trauma or uveitis can induce secondary glaucoma. This latter is a common complication of uveitis affecting 20% of patients. Some ocular inflammations associated with secondary glaucoma that should be considered in the context of uveitic glaucoma are the herpetic keratouveitis, Fuch's heterochromic iridocyclitis, or the Posner-Schlossman syndrome [22]. It has also been reported that ocular comorbidity such as glaucoma or other surgery treatments following intraocular lens implantation may contribute to its opacification [23]. Additionally, higher prevalence of retinal diseases (DR, AMD) in glaucoma patients suggested a similar pathological process that needs further consideration [24].

Other retinopathies, such as the inherited retinal degenerative diseases, affect millions of people around the world, displaying several degrees of visual impairment and irreversible vision loss, with retinitis pigmentosa, choroideremia, juvenile retinoschisis, Stargardt disease, Usher disease, or Leber congenital amaurosis being the most frequent. Likewise, retinitis pigmentosa remains the leading cause of inherited blindness, with approximately 2 million people affected worldwide. Multiples genes have been identified and their mutation may result in the corresponding phenotype to retinitis pigmentosa. However, there is lack of knowledge on the molecular mechanisms involving the disease that exhibits a progressive and irreversible nature leading to continuous decline of the visual field and vision. Emerging treatments hopefully include gene therapy, stem cells, and electronic devices to restore vision [25]. Common eye comorbidities to retinitis pigmentosa are glaucoma and cataracts extremely contributing to the visual disability of the affected individuals [26].

Strategies to achieve a precocious diagnosis and to accurately plan the therapy of patients with ocular comorbidities may help in avoiding dangerous complications and visual loss.

## 3. Systemic Disorders and the Eye

Many diseases can directly or indirectly damage the eyes and vision, while other diseases possess associated ocular signs/symptoms and visual impairment. All of these have distinct mechanisms of action. For a better understanding, this section has been structured into two parts: systemic diseases with ocular manifestations and systemic syndromes and the eyes. Both sets of issues are exposed below.

### 3.1. Systemic Diseases with Ocular Manifestations

Main systemic disorders which may affect our eyes include endocrine and/or metabolic diseases, inflammatory and immune response processes, infections, hematological, cardiovascular, and cerebrovascular disorders, cancer, skin illnesses, and congenital/hereditary conditions. A summary of the processes that can affect the eyes and vision is reflected as follows.

Systemic diseases with eye involvement include the following:

 Hematologic and lymphatic diseases.
 Cardiovascular/cerebrovascular diseases.
 Gastrointestinal/nutritional disorders.
 Metabolic/endocrine disorders.
 Musculoskeletal pathologies.
 Pulmonary diseases.
 Renal disorders.
 Systemic viral infections.
 Systemic bacterial infections.
 Systemic protozoal infections.
 Systemic fungal infections.
 Systemic cestode and nematode infections.
 Dermatologic pathologies.
 Phacomatoses.
 Collagen diseases.
 Multisystemic autoimmune diseases.
 Granulomatous diseases.
 Immunosuppressive agents used in management of eye disease.
 Ocular complications of certain systemically administered drugs.
 Neoplastic diseases with ocular metastases.
 Vitamins and eye diseases.
 Miscellaneous systemic diseases with ocular manifestations.
 Heritable connective tissue diseases.
 Hereditary metabolic disorders.
 Genetic syndromes.

#### 3.1.1. Major Pathologies with Ocular Involvement

Some particular diseases are openly acknowledged to disturb the visual system, to a degree that the ocular manifestation may be used to accurately confirm the most complete diagnosis, as well as monitoring the appropriate therapy, such as in cases of diabetes mellitus (DM), hypertension blood pressure (HBP), hyperthyroidism, sarcoidosis, tuberculosis, arthritis, psoriasis, scleroderma, or systemic infections. The most relevant ones are explained in detail in this subsection.

Progression of DM of any type causes the diabetic eye disease that includes several sight-threatening ocular disorders, with the DR and diabetic macular edema (DME) being the most important that in the course of the disease may lead to visual impairment and blindness (Figure 1). In fact, both disorders, DR and DME, are leading causes of vision loss among working-aged adults (20–70 years) [27, 28]. Our understanding of the precise mechanisms by which DM induces DR and/or DME remains incomplete. A wide range of ocular pathologies are also associated with DM, such as cataracts, glaucoma, and optic neuropathy. Other ocular associations of DM distinct from DR are the anterior ischemic optic neuropathy, diabetic papillopathy, and extraocular muscles disorders [29]. Regarding these pathologies, studies suggest that up to 25% of patients with anterior ischemic optic neuropathy have a history of DM. Furthermore, diabetic papillopathy is characterized by acute disc edema and mild vision loss appearing suddenly in the course of diabetes. Also, extraocular motility disorders and diplopia occur in 25–30% of diabetic patients aged 45 years and older. Ocular diseases in which DM can be considered among the etiology also include the retinal vein/arterial occlusion or some corneal disorders. Furthermore, distinctive features have extensively been described during the ophthalmic surgery in diabetics. This topic has been reviewed by the Pan American Collaborative Retina Study Group (PACORES) in a study designed to evaluate the visual and anatomical outcomes after cataract surgery in diabetic patients with different intraoperative therapeutic strategies [30]. It is essential to promote early detection, timely and accurate treatment, and appropriate control of the clinical manifestations of the diabetic eye disease in order to prevent blindness in diabetics.

**Figure 2:**
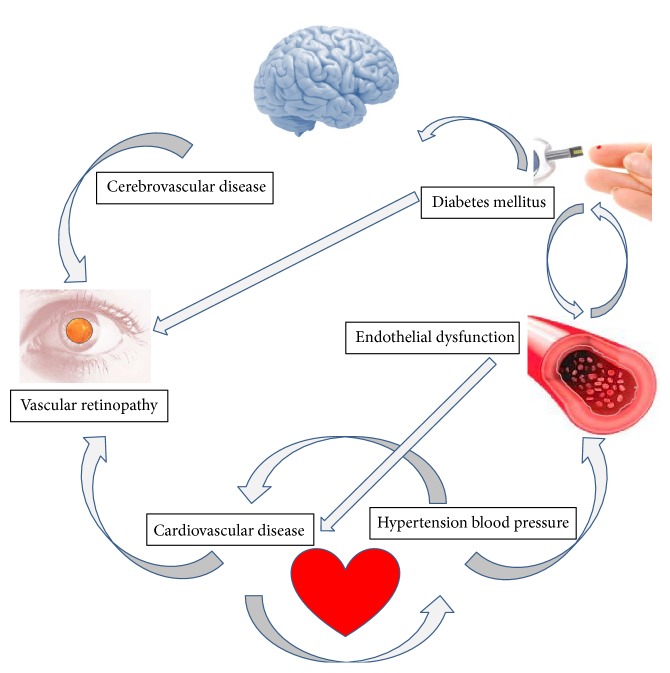
Pathogenic mechanisms of the vascular retinopathies.

Retinal vascular changes can be the starting point of an asymptomatic HBP patient (Figure 2). However, in the course of the disease, both the acute and chronic hypertensive changes may display in the eyes severe abnormalities induced from the existence of a malignant HBP, or chronic changes resulting from long-lasting HBP. Retinal, choroidal, and optic nerve changes can be seen in different stages of the systemic disease widely known as the acute hypertensive retinopathy (or choroidopathy or neuropathy), as well as the chronic hypertensive retinopathy (or choroidopathy or neuropathy) [31]. All these processes are the result of adaptive changes and progressive degenerative damage to the arterial and arteriolar circulation caused by the HBP. It has been stated that major risk factors for the initiation or progression of hypertensive retinopathy are age, duration of hypertension, and systolic blood pressure levels. Severe degree of hypertensive retinopathy correlated with serious blood pressure concerns and the highest risk for stroke, as well as kidney and heart disease, while low levels of hypertensive retinopathy did not correlate with cardiovascular risks [32]. Moreover, the HBP predisposes patients to other eye disorders, such as retinal vascular diseases (central/branch retinal artery or vein occlusion, macroaneurysms, neovascularization, vitreous hemorrhage, epiretinal membrane formation, tractional retinal detachment, chronic papilledema, and optic atrophy) [33]. It is important to consider that HBP is an important contributing factor to DR leading to more advanced DR progression rates (see Figure 1) [34].

Retinal vascular changes appear in parallel with the pathological changes occurring in the coronary circulation. It has been described that retinal arteriolar narrowing was associated with reduced myocardial perfusion as determined by cardiac magnetic resonance imaging [35]. Other studies dealing with this topic reported that retinopathy signs positively correlated with coronary artery calcification (measured on cardiac computed tomography scanning) in a dose response manner, with more severe lesions associated with worse coronary artery disease on angiography [36, 37]. As a result of all these data, there is enough evidence to confirm that alterations in the retinal microvasculature may be pivotal indicators of the pathologies linked to vascular structure of the coronary microcirculation (Figure 1).

Patients suffering from scleritis and/or uveitis always have to be worked up for underlying systemic causes. Assessment for mucosa or skin lesions, arthritis, or infections may be carried out in each scleritic or uveitic patient. Uveitis associated diseases include syphilis, tuberculosis, ankylosing spondylitis (HLA-B27), or sarcoidosis. Rheumatoid polyarthritis, systemic lupus erythematosus, and systemic vasculitis were the most frequent associations with posterior scleritis that is commonly linked to other systemic diseases (40% of the cases) [38]. A multisystemic inflammatory process (*T-cell-mediated autoimmune disorder directed against melanocytic antigens*) known as the Vogt-Koyanagi-Harada syndrome, characterized by the panuveitis and serous retinal detachment, against a background of diverse neurologic and cutaneous manifestations, can be early detected and aggressively treated to prevent visual loss [39].

Respiratory disorders can also have an impact on the eyes. With this in mind, prevalence of obstructive sleep apnea syndrome has been found to be high in patients with nonarthritic anterior ischemic optic neuropathy and also in glaucomatous individuals [40]. Based on this and similar descriptions, performing polysomnographies in the affected patients has been recommended. Following this topic, inhaled corticosteroids (high doses/long-lasting treatments) are used by patients with chronic obstructive pulmonary diseases [41]. As a consequence of this therapy, a wide variety of ocular and systemic effects have been described such as cataracts, glaucoma, DM, HBP, pneumonia, and osteoporosis. Also, sleep apnea has been related to glaucoma progression [42]. Therefore, it is necessary to provide the most appropriate tools for monitoring these patients in order to prescribe proper treatment and preserve visual functions.

Regarding the kidney diseases, a special risk for specific ocular comorbidities such as dry eyes, uveitis, cataracts, and glaucoma is noticeably high in patients with chronic renal failure, as reported in a recent study including 9,149 patients and 27,447 matched controls (age 40–98 years) from the Taiwan study group [43].

#### 3.1.2. Main Ocular Manifestations Induced by Systemic Disorders

In many occasions, the eye signs and symptoms are the first visible or the most evident manifestation of other important systemic problems, including infections, traumatisms, neurodegenerative and mental disorders, thyroid dysfunction, autoimmune diseases, pharmacological drugs, and toxic substances.

Therefore, a systematized ophthalmic examination is essential for managing both the ocular pathology and the underlying systemic disorder.

In an infectious background, it has been reported that 60% of patients with acquired immunodeficiency syndrome display ocular disorders, which increases up to 90% in necropsies. Most common eye disorders in AIDS are cytomegalovirus retinitis and retinal microvasculopathies [44]. Precisely, emerging and resurging viral infections strongly represent a public health problem worldwide. Among them, dengue fever, chikungunya, Ebola virus, enterovirus, hantavirus, Henipavirus, influenza virus A (H1N1), Japanese encephalitis, Kyasanur forest disease, rickettsioses, Rift Valley fever, and West Nile fever may result in different ocular pathologies, such as chorioretinitis, vitreoretinitis, retinal vasculitis, optic neuropathy, retinal hemorrhages, or any other ocular inflammatory condition usually involving all the eye components [45]. Lyme neuroborreliosis is a disease caused by the tick-borne spirochaete* Borrelia burgdorferi* involving central nervous system and neurosensory organs, among others. The most frequent clinical symptoms observed are headaches (71%), vertigo (44%), meningeal symptoms (22%), and neurological paresis (27%) (including facial palsy, 23%). However, neuroretinitis has also recently been described in patients with Lyme disease [46].

Traumatic events are usually reflected in our eyes. Importantly, our eyeballs act as an open window reflecting obscure situations that may undoubtedly help physicians to detect silent damage to children and adults, such as in the cases of abusive head trauma in battered wives or in the shaken baby syndrome [47]. Furthermore, it has been reported that patients with posttraumatic stress disorder or depression have differences in dry eye symptoms and signs compared to a population without this condition [48].

It has been recently emphasized that psychological distress and depression are frequent comorbidities in glaucomatous individuals [49], as well as AMD patients [50]. Likewise, optic nerve degeneration in glaucoma patients has been found to frequently coexist with Alzheimer or Parkinson diseases and other neurodegenerative disorders [51, 52].

To keep on the prevalence of selected systemic comorbidities in patients with primary open-angle glaucoma (POAG), the most prevalent glaucoma type, Lin et al. performed a nationwide, case-control study using an administrative database (76,673 POAG patients and 230,019 healthy subjects) including 31 medical comorbidities selected from the Elixhauser Comorbidity Index [53]. The authors reported that the prevalence difference of the glaucomatous patients with respect to the controls was 3% or higher for hypertension, hyperlipidemia, stroke, diabetes, liver disease, and peptic ulcer [54]. Often these glaucomatous individuals are completely full with other diseases, including DM, HBP, and CVD. Minor comorbidities with glaucoma are thyroid dysfunction, Alzheimer or Parkinson's disease, anxiety, depression, and stroke. It has to be considered that these patients have many more things going on simultaneously with glaucoma; because of this, the scope of concerns with these individuals seriously increases in comparison with others.

Thyroid orbitopathy also named Graves' orbitopathy is an autoimmune disorder associated with thyroid dysfunction that is manifested with typical self-limiting eye and adnexa signs and symptoms. Currently, the pathogenesis and effective treatment for this disease remain elusive. Manifestations of thyroid orbitopathy include eyelid retraction (affecting 90–98% of patients), lagophthalmos caused by incomplete eyelid closure, exophthalmos (eyeball proptosis and poor blinking), and extraocular muscles dysfunction which cause diplopia. Excessive eye exposure leads to increased tear evaporation and DEDs as well as superior limbic keratoconjunctivitis [55]. The coexistence of thyroid orbitopathy and myasthenia gravis with more severe ocular repercussion has also been reported [56]. The goal of managing patients with Graves' disease is the control of the thyroid state. Ophthalmologists may act in combination with endocrinologists, neurologists, and/or maxillofacial specialists to improve eye care.

Pursuing with the factors leading to the most relevant ocular manifestations induced by systemic disorders, the aging process has to occupy a preferential place. The World Health Organization estimated that the age-related eye disorders and visual impairment affect over 372 million older adults worldwide. Therefore, elder people suffering from comorbid conditions in the context of visual impairment constitute an additional problem because they importantly suffer impaired quality of life, a greater risk of falls (and the so-called “fear of falling”), faster cognitive decline, and a higher risk of accelerated aging and/or premature death as compared to individuals without visual affection [57]. There are four major age-related eye diseases: cataracts, glaucoma, DR, and AMD. Here, we will show the AMD facts related to the topic of this review, because the other diseases have yet been previously considered. The AMD is the leading cause of severe vision loss in people aged 60 years or more. Wong et al. have estimated the global prevalence of the disease and its burden projection for 2020 and 2040 and the results confirmed that the projected number of people with AMD (any clinical type) in 2020 is 196 million, increasing to 288 million in 2040 [58]. Having these data in mind, it is essential to review the systemic disorders that may worsen the vision-related quality of life of the affected patients. Several general pathologies have been associated with AMD such as HBP, CVD, cerebrovascular disease, dyslipidaemia, chronic kidney disease, and neurodegenerative disorders; some of them have been reviewed above. Currently, increasing evidence points to the fact that AMD patients are at risk of stroke. Interestingly, it has been suggested that AMD is an ocular manifestation of systemic disease processes [59].

It is well known that every pharmacological substance (topically or/systemically administered) can induce unfavorable effects (eyelids, conjunctiva, cornea, lens, iris, ciliary body, retina, optic nerve, and the extraocular muscles), even when utilized according to standard protocols. Among the medicaments that may cause ocular toxicity and vision loss are chloroquine/hydroxychloroquine, thioridazine, chlorpromazine, tamoxifen, ethambutol, isoniazid, fluoroquinolones, and monoclonal antibody therapy [60]. It has also to be seriously considered that craniofacial and eye developmental abnormalities can be induced by the use of these drugs during pregnancy, as well as by the alcohol or psychostimulants abuse by the pregnant women [61, 62].

The ocular manifestations of the systemic diseases have to be managed by the ophthalmologists with the cooperation of the professionals involved in the related medical specialties. Also the utilization of new technological devices can help to achieve an early diagnosis of the affected patients. These new tools for ophthalmic examination require high-level technically skilled and knowledgeable users, as in the cases of the latest developments such as optical coherence tomography (*OCT angiography, en face OCT*), Scheimpflug imaging, scanning laser ophthalmoscope, ultra-wide-field imaging, microperimetry, multifocal electroretinogram, Doppler imaging, or the ocular ultrasound and magnetic resonance imaging. With these new techniques, an accurate diagnosis and proper therapy monitoring can be reached to better manage patients with systemic disorders and eye pathologies, mainly in cases of corneal or retinal damage [63–65].

### 3.2. Genetic Syndromes and the Eyes

Genetic syndromes are disorders caused by changes or mutations in the DNA which alter the synthesis or function of proteins. This usually involves major changes in the physical and behavioural development of the affected patient. In many cases, the alterations include eye disorders such as cataracts, glaucoma, myopia, or retinopathies, as well as craniofacial and ocular malformations.

Down syndrome (a trisomy of chromosome 21) and eye disorders coexist in 60% of all cases, with the following being the most frequent: strabismus, astigmatism, cataracts, or myopia [66]. The second genetic cause of mental retardation in the world, the fragile X syndrome (or Martin-Bell syndrome), is caused by a mutation in the regulatory region of the FMR1 gene on the X chromosome. Strabismus, myopia, and hypermetropia are commonly found in the affected patients [67, 68]. In cases of the Angelman and the Prader-Willi syndromes (caused by mutations in chromosome 15), strabismus is usually diagnosed. Bardet-Biedl syndrome is a rare autosomal recessive disease with very different manifestations, such as obesity, polydactyly, and mental retardation. Retinitis pigmentosa is one of the major features of this disorder [69]. Marfan syndrome is an autosomal dominant disease caused by a mutation in the FBN1 gene on chromosome 15, which causes changes in the function of fibrillin. The classic Marfan is the most common clinical type, with the following being the accompanying eye disorders: retinal detachment, cataracts, lens displacement, and glaucoma [70]. Other rare GS also include ocular alterations, including Cri-du-chat syndrome (myopia, optic atrophy), Lowe syndrome (congenital cataracts, infantile glaucoma) [71], Marinesco-Sjögren syndrome (cataracts) [72], and Axenfeld-Rieger syndrome (50% develop glaucoma) [73].

In summary, the genetic syndromes are disorders affecting different organs that induce a high variety of symptoms and conditions. There are many ophthalmic features associated with these disorders, and very occasionally, suspicion of the genetic syndromes is raised by first presentation with ocular problems, with the following being the most frequent: strabismus, important refractive errors, and cataracts. The following have to be considered less frequently: myopia, retinal degeneration, and glaucoma.

*The Need for a Comprehensive Evaluation of Eye Comorbidities and Systemic Disorders*. A wider knowledge on the eye manifestations of systemic diseases as well as the ocular comorbidities can help to early diagnose a specific disorder, slow the progression, and/or prevent visual impairment or blindness in patients suffering serious eye complications.

Thus, a thorough ocular evaluation should include a precise anterior eye segment and media and dilated fundus examination, and when indicated, fluorescein angiography, OCT, visual field, and/or radiologic probes should be performed in patients suspected of being affected by an eye manifestation of systemic disease or an ocular comorbid condition.

It has to be considered that, in some cases, the eyes can show signs of an internal disorder before the disease progresses and becomes a more serious problem. The key is to understand what is happening as soon as possible to avoid severe complications for the health and vision. A wide spectrum of observable eye changes and visual variations can be recognized either by the patient himself/herself or by the physician. However, a handful of ophthalmological signs can certainly point to a systemic disorder. Among the external signs are the following: (1) specific conjunctival or scleral hyperemia or violet areas that do not respond to therapy, (2) bilateral palpebral eczema or swelling without secretion, (3) spots and pigmented (or depigmented) areas growing or changing, (4) episodes of partial or complete visual loss with/without aura, (5) external muscles intermittent of progressive alterations with ocular misalignment, eye strain, and diplopia, and (6) malposition of the upper eyelid with/without enophthalmos or protrusion of the eyeball. Most relevant internal signs include the pupillary abnormalities, aqueous humor or vitreous body changes, retinal arteries and veins alterations, and the presence of two or more of the following: retinal spots, pigmented zones, exudates, hemorrhages, atrophic areas, papillary swelling, and choroidal neovascularization.

The ocular comorbidities as well as the eye-related systemic disorders increasingly strain healthcare sectors and societies worldwide, especially within the aging population.

Most patients are primarily managed by general physicians and advanced practice nurses. A precise early diagnosis is needed with appropriate protocols in order to avoid severe complications, visual impairment, and blindness. For successful global and eye/vision care, outstanding new strategies on the basis of an interdisciplinary team have to be established with the main goal of introducing a variety of quality improvement interventions that can achieve better results in the clinical practice and health systems [74–77]. Appropriate training and effective communication among the primary care physicians, specialists and subspecialists, nursing, and other health care professionals, as well as the collaboration of patients and their family members and caregivers, are critical for ensuring the intervention effectiveness [78–80]. Practical elements for improving the most accurate diagnosis and management regarding the ocular comorbidities as well as systemic disorders with eye repercussion have to be highlighted, which can be implemented with relatively easy plans and little financial inputs [81, 82].

For the past fifteen years, our clinical and experimental research team has contributed to the knowledge and skills about the ocular comorbidities and the eye manifestations related to systemic disorders. The main challenge is to share the personal expertise with each other, to increase cooperativity in order to prevent blindness.
